# Supplementary material for: Are carbon emissions trading and green financial instruments synergistic? -Comprehensive quantitative research based on content analysis
Source: PLoS One. 2024 Mar 7;19(3):e0298601. doi: 10.1371/journal.pone.0298601 (PMC10919601; doi:10.1371/journal.pone.0298601)
Supplement: S1 Data — (ZIP) [file pone.0298601.s001.zip › Data/地级市Z_F工作报告.zip!/╕ú╜¿/╧├├┼╩╨/└┤╫╘╣1⁄2╓┌║┼í╛┬φ┐╦╩2╛▌═°í┐_2018.docx]

各位代表:

　　现在，我代表市人民政府向大会报告工作，请予审议，并请市政协委员提出意见。

　　一、2017年工作回顾

　　2017年，我们以迎接党的十九大和学习宣传贯彻十九大精神为动力，以习近平新时代中国特色社会主义思想为指导，在省委省政府和市委的坚强领导下，着力稳增长、促改革、调结构、惠民生、防风险，各项工作全面进步，质量效益持续提升，经济社会保持平稳健康发展。完成地区生产总值4351.2亿元，增长7.6%；固定资产投资2381.5亿元，增长10.3%；财政总收入1187.3亿元，增长9.6%，其中，地方级财政收入696.8亿元，同口径增长11%；城乡居民人均可支配收入增幅与经济增长基本同步；居民消费价格上涨2%；完成年度节能减排任务。

　　一年来的主要工作和成效:

　　（一）厦门会晤筹备和服务保障工作圆满完成

　　厦门会晤是2017年我国主办的最重要的主场外交活动之一。我们坚决贯彻习近平总书记重要指示精神，在国家有关部委、省委省政府和市委坚强领导下，认真落实节俭务实少扰民各项措施，充分利用现有场馆设施，做到能借就不租、能租就不买，会晤期间尽量不放假不停工不停课，出色完成了各项工作。习总书记深情赞誉厦门是高素质的创新创业之城、高颜值的生态花园之城，全市人民倍感振奋。会晤的成功举办增强了全市人民的获得感。把筹备工作与灾后恢复重建、文明城市创建有机结合，全面提升城市品位，更新建筑物立面9069栋，建成夜景照明工程2038处，种养绿植近100万株，改造道路216公里，缆化通讯管线188公里、电力管线33公里，有效改善了人居环境。狠抓城市治理，全面开展基础信息大排查，强化群防群治，平安厦门实现质的提升。会晤的成功举办汇聚了积极向上的正能量。把筹备工作作为锤炼干部、展示作为的重要平台，层层压实责任、强化正向激励，形成了一整套严谨务实高效的工作机制。广大干部职工恪尽职守、连续奋战，锻造了实干精神和过硬作风。广大市民群众积极支持、无私奉献，用实际行动展现了城市深厚的文明底蕴，赢得创建全国文明城市五连冠，为新时代新发展注入了强大动力。

　　（二）产业转型升级成效明显

　　高度重视发展实体经济，通过加强经济运行调度、编制产业链招商总图、分类扶持龙头企业和产值增长大户、挂钩帮扶困难企业等一系列措施，推动了产业结构进一步优化。实施“中国制造2025”、促进工业稳增长和转型升级获国务院表扬，进入全球可持续竞争力百强。创新驱动能力不断增强。火炬高新区获批建设国家“双创”示范基地。新增国家高新技术企业200家左右，规上高新技术产业增加值占规上工业67.9%。国家自主创新示范区推出69项创新事项，落地产业科技项目191个，新建及认定公共技术服务平台、工程技术研究中心、重点实验室54个。全社会研发投入占GDP比重达3.11%，每万人拥有有效发明专利为全国2.3倍，入选国家十大知识产权强市创建市。累计设立众创空间165家、在孵团队2759支。国家“千人计划”人才增至102人，40人入选国家创新人才推进计划。先进制造业不断壮大。规上工业增加值增长8.1%，工业利润总额增长22.4%。电子、机械两大支柱产业产值占规上工业68.2%。开工建设亿元以上产业项目331个、总投资3734亿元，在谈工业项目90项、总投资847亿元。新认定国家级工业设计中心3家，总数占全省56%，新增国际、国家、行业标准153项。战略性新兴产业加快发展。集成电路形成覆盖设计、制造、封装、测试的全产业链，产值居全国第5位。软件信息服务业营收增长16.4%，5家企业入选中国互联网企业百强。生物医药港跻身全国九大生物医药产业园，全球首个戊肝疫苗进入世界卫生组织采购体系。新材料产值507亿元，增长23%，石墨烯、碳化硅等开始量产。文化创意产业总收入907亿元，增长17.5%。海洋经济总产值2295亿元，增长10.7%。现代服务业加快提升。服务业增加值增长7.9%，占GDP比重57.7%，金融等四大行业对服务业贡献率78.4%。内贸流通体制改革经验在全国推广，社会消费品零售总额增长12.7%，限额以上网络零售额增长81.3%。旅游接待7830.5万人次，旅游总收入1168.5亿元，分别增长15.7%和20.7%。举办展览215场、外来商务会议超过8000场，获评中国旅游休闲示范城市、最佳会奖营销目的地，进入世界会展城市50强。金融业增加值增长8%，本外币存款余额首次超万亿元，银行不良贷款率1.47%，比降0.4个百分点。新批境内上市公司11家、新三板挂牌企业37家，总数均为全省第一。物流产业总收入1100亿元，增长8.9%。服务外包执行金额增长38%，太古飞机入选中国服务外包领军企业十强。都市现代农业稳步推进。农业产业集群营收739亿元，新建74个设施农业项目、农业“五新”成果示范基地和标准化生产基地。基本完成农村土地承包经营权确权登记。

　　（三）发展动力活力显著增强

　　开展新一轮降成本优环境专项行动，狠抓改革攻坚和开放合作，获评全国城市信用建设示范市和创新奖，国际营商环境经第三方评估居全国前列。供给侧结构性改革取得实质进展。实施营改增、下调社会保险费率、取消47项涉企收费等政策措施，为企业减负超过250亿元。深化“放管服”改革，取消或下放行政审批事项72项，减少各类证明材料230项，80%以上审批服务实现“一趟不用跑、最多跑一趟”，电子政务发展指数居全国第4位。新增商事主体11.9万户，增长22.8%。民间投资增长20.4%。市属国企资产总额增长18.8%，利润增长43.8%，建发、国贸跻身世界500强。自贸试验区建设成效明显。围绕打造“三区一堡”，加快推进投资自由化、贸易便利化、产业高端化，累计推出创新举措298项，其中全国首创49项、在全市复制推广214项。在工程建设和市场准入领域开展行政审批制度改革试点，大幅压缩时限、提高效率。建成国际贸易“单一窗口”2.0版，成为全国第2个加入亚太示范电子口岸网络的成员口岸。关检一站式查验平台+监管互认被评为最佳实践案例。中欧（厦门）班列通达9个国家13个城市，累计发运221列，成为中欧安智贸首条铁路航线试点。打造16个重点平台，获批并投用汽车整车进口口岸，成为全国第三大进口酒口岸。获批航空区外保税维修政策，航空维修业产值占全国1/4，增长10%。累计设立融资租赁企业288家、开展52架飞机租赁业务，首次开展大型设备、船舶租赁。对外交流合作更加紧密。实际使用外资160.1亿元，增长11.2%，规模居全省首位。外贸进出口额5816亿元，增长14.3%，其中进口增长28.4%，出口增长5.2%，扭转了出口连续下滑态势。积极落实“一带一路”倡议，对“海丝”沿线国家贸易额2083亿元、增长12.8%，投资额22亿美元、增长70%，新增合作项目30个，设立全国首支地方政府主导的“海丝”投资基金。厦洽会、国际海洋周等活动影响力进一步增强。对台战略支点作用更加凸显。围绕促进两岸融合发展，深入实施综合配套改革，货物、服务、资金、人员流动更加便利。新批台资项目668个，实际使用台资增长12.6%。初步建成集货币清算、跨境贷款、现钞调运为一体的两岸银行货币合作平台。对台海运快件增长34.9倍。厦金航线周末及节假日增加船班实现常态化，出入境旅客161.4万人次。成功举办第九届海峡论坛和工博会、文博会、旅博会等40多项重大活动。

　　（四）城市承载力宜居度持续提升

　　大力拓展城市空间，着力完善基础设施、优化人居环境，现代化城市建设迈出新步伐。新城基地建设加快。开工项目232个、在建401个、竣工110个，完成固投826亿元，电力、供水、燃气、学校、医院等配套加快完善。集美新城累计引进企业1549家，产城融合进一步提速。环东海域新城和现代服务业基地206个公建项目全面推进，滨海旅游浪漫线加快建设。马銮湾新城146个公建项目进展顺利，三横三纵骨干路网实现通车。同翔高新技术产业基地起步区一期、二期框架初步形成。基础设施更加完善。成为全国最高等级国际性综合交通枢纽。翔安机场填海造地、交通市政等配套工程稳步实施。国际航运中心加快建设，国际中转箱量增长72%，集装箱吞吐量突破1000万标箱。地铁1号线开通运营，2、3、4、6号线累计开工64个车站，区间掘进37公里，厦门迈入地铁时代。福厦高铁厦门段正式开工建设。基本完成同集路改造，建成灌新路、集灌路等一批工程，“两环八射”外环基本贯通。城市管理更加精细。创新城管执法机制，拆除“两违”672万平方米，户外广告、流动摊贩、露天烧烤、不文明施工等专项整治成效明显。稳步推进25项交通疏解工作，打通6条断头路，整治20处安全隐患路段和一批学校医院周边堵点，新增优化公交线路97条，港湾式改造公交站点38座，新增公共停车位7500个，清理不合规网约车、规范共享单车取得初步成效。生活垃圾分类全面推行，覆盖全市主城区90%小区和所有公共区域，知晓率超过90%、参与率超过80%，做法和经验成为全国范例。城乡环境持续改善。基本完成19项生态文明体制改革任务。空气质量综合指数在全国74个重点城市排名第4。长泰枋洋水利枢纽一期工程建成通水，莲花水库建成蓄水，我市供水水源实现由“一江两库”到“两江四库”。全面推行河长制，基本消除6处水体黑臭现象，实现9条溪流465公里河道养护全覆盖，最严格的水资源管理制度考核名列全省第一。完成营造林4.1万亩，海沧湾整治成为蓝色海湾国家示范工程。严打非法采砂洗砂，修复岸线14.5公里。建成500千伏厦门变电站三期等电力设施。完成海沧、同安门站建设，实现管道燃气双气源保障。在全国率先确立海绵城市建设管理标准体系，地下综合管廊绩效评价全国第一。

　　（五）民生改善成效日益凸显

　　坚持为民惠民理念，加强和创新社会治理，着力补齐民生短板，群众获得感、幸福感、安全感持续增强。社会事业取得积极进展。鼓浪屿申遗成功，习近平总书记作出重要指示，为文化遗产保护指明了努力方向、提供了根本遵循。新改扩建中小学项目60个，建成中小学校20所，新增学位2万个；开建公办幼儿园22所，新增学位6500个。2.2万名外地务工人员随迁子女通过积分入学。国际学校建设加快。教育质量稳步提升，高考成绩突出。居民主要健康指标居全国前列。复旦大学中山厦门医院建成投用，新增床位800张。北京中医药大学与厦门中医院开展深度合作，成立厦门分院。厦门特色分级诊疗和家庭医生签约服务模式向全国推广。医疗救治应急指挥平台投入运行，国家健康医疗大数据中心及产业园建设扎实推进。全运会金牌数全省第一，30项国际和国家级赛事成功举办，厦门马拉松赛实现国际田联路跑金牌赛事十连冠。高甲戏《大稻埕》获国家五个一工程奖，故宫鼓浪屿外国文物馆正式开馆。社会保障日趋完善。新增就业18.6万人，发放2.2亿元企业稳岗补贴，支持3930家企业开展技能培训。社会保险参保人数稳步增加，巨灾保险顺利开展。社会救助和保障标准与物价上涨挂钩联动，城乡低保和特困人员基本生活标准分别提高到每人每月720元和1080元。企业退休人员月人均基本养老金提高到3422元，最低工资标准提高到1700元，均居全国前列。困境儿童分类保障制度更加完善。居家养老实现城区全覆盖、农村基本覆盖，医养结合试点不断深化，养老机构床位中护理型占比达83.7%。开工及续建保障性住房4.1万套，在建总量接近前十年建成总和。按照区位好、配套好、品质好、环境好、规模大的标准，规划建设4个保障房地铁社区，一期项目全面开工。出台限购限贷限售政策，房价过快上涨势头得到有效遏制。社会治理水平持续提升。社区网格化管理服务不断升级，持证社工占全省46%。食品药品安全追溯系统投用，全国食品安全示范城市创建中期评估名列第一。在生态环境优良的南平等地建立万亩优质副食品供应基地，已正式向我市供货。安全生产责任制落实有力，标准化建设持续完善，安全生产大检查和重点行业领域专项整治全面推进，2281家违规使用醇基燃料餐饮场所整改清零。刑事警情下降42.8%，重点信访积案办结率大幅提升。军民融合深度发展，国防动员创新推进，实现省级双拥模范城（区）“满堂红”四连冠。36项为民办实事项目全面完成。妇女、儿童、青年、老年、民族、宗教、侨务工作继续加强，残疾人、红十字、慈善、人防、气象、档案、地方志等工作取得新进步。

　　（六）政府自身建设全面加强

　　牢固树立“四个意识”，严守党的政治纪律和政治规矩。深入推进“两学一做”学习教育常态化制度化，严格落实中央八项规定精神和国务院约法三章，全市“三公”经费支出持续下降。自觉接受人大法律监督、工作监督和政协民主监督，认真办理市人大代表建议298件、市政协提案483件，办复率均为100%，满意率分别为99%和95%。提请市人大常委会审议法规草案5件，制定市政府规章4件，废止和宣布失效市政府规范性文件50件，立法推进社会文明建设走在全国前列，获评法治政府建设典范城市。加强政府系统党的建设，认真落实意识形态工作责任制，修订市政府工作规则，出台市政府重大行政决策规定。加大行政监察和审计监督，狠抓巡视巡察发现问题整改，查处追究41家单位92个效能问题、问责150多人，推动作风建设实现长效化。

　　各位代表！过去的一年，我们勠力同心、真抓实干，攻坚克难、稳中求进，在决胜全面建成小康社会进程中迈出坚实步伐。这些成绩的取得，是在市委坚强领导下，在人大代表、政协委员有效监督和大力支持下，全市人民齐心协力、艰苦奋斗的结果。在此，我谨代表市人民政府，向全市人民，向全体人大代表和政协委员、各民主党派、工商联、各人民团体、无党派人士、离退休老同志和社会各界人士，向中央和省驻厦单位、驻厦部队、武警官兵，向来厦的投资者和劳动者，向关心支持厦门发展的港澳同胞、台湾同胞、海外侨胞和国际友人，表示衷心的感谢和崇高的敬意！

　　同时，我们也清醒地看到，发展中还存在不少困难和问题，主要是:地区生产总值等指标未能达到年初预期，房地产对经济社会发展影响不容忽视；发展后劲有待增强，实体经济发展水平有待提高，尤其工业投资总量偏少、比例偏低，出口增长比较乏力；发展不平衡不充分问题尚未解决，岛外新城建设仍需大力推进，住房、教育、医疗等民生领域还存在不少短板，社会治理等还有薄弱环节；政府职能转变和体制机制创新还需加大力度，部分干部担当意识有待增强，党风廉政建设和反腐败斗争任务依然艰巨。对这些问题，必须采取有力措施，认真加以解决。

　　二、2018年主要工作

　　今年是贯彻党的十九大精神的开局之年，是改革开放40周年，是决胜全面建成小康社会、实施“十三五”规划承上启下的关键一年，我们要以更高标准、更严要求、更实作风做好全年各项工作，努力为全国全省大局作出新的更大贡献。

　　政府工作总体要求是:全面贯彻落实党的十九大精神，以习近平新时代中国特色社会主义思想为指导，深入贯彻落实习近平总书记对福建、厦门工作的重要指示精神，坚持稳中求进工作总基调，坚持新发展理念，紧扣社会主要矛盾变化，按照高质量发展的要求，统筹推进“五位一体”总体布局和协调推进“四个全面”战略布局，坚持以供给侧结构性改革为主线，把高质量发展与实现赶超有机统一起来，统筹推进稳增长、促改革、调结构、惠民生、防风险各项工作，大力推进改革开放，持续强化创新驱动，推动质量变革、效率变革、动力变革，奋力开创新时代“五大发展”示范市建设新局面，进一步建设高素质高颜值的现代化国际化城市，以实际行动当好新时代中国特色社会主义排头兵。

　　经济社会发展主要预期目标为:地区生产总值增长8.5%左右，固定资产投资增长15%，财政总收入和地方级财政收入分别增长8%和8.2%，社会消费品零售总额增长10%，城镇居民和农村居民人均可支配收入分别增长8%和8.5%，居民消费价格涨幅控制在3%左右，完成国家和省下达的节能减排任务。

　　重点做好七个方面工作:

　　（一）坚定不移推进质量变革，全力建设高素质创新创业之城

　　坚持质量第一、效益优先，坚持链群齐抓、赶转并重，深入实施千亿产业链（群）培育工程，加快建设实体经济、科技创新、现代金融、人力资源协同发展的产业体系。一是大力实施创新驱动发展战略。加快建设国家自主创新示范区，统筹推进“双自联动”，打造区域科技创新中心。全力推进“19+20”创新平台项目，新增国家高新技术企业200家、科技小巨人企业150家。深入实施质量强市战略，不断深化标准、质量、品牌、信誉一体化建设。加快建设科技金融结合试点城市、知识产权强市，加强知识产权创造、保护、运用，提高科技成果转化水平。二是大力发展先进制造业。围绕增强制造业核心竞争力，加快结构优化和转型升级，提升集聚化、高端化、智能化水平。加强技改奖补等扶持政策系统集成，重点支持优势产业发展壮大，力争完成工业投资490亿元。推进一批产业链配套延伸项目，力争计算机与通讯设备、机械装备产业链产值分别突破千亿。促进太古飞机维修基地搬迁等15个项目开工，加快长塑薄膜、盈趣产业园等70个续建项目建设，力争欧米克等30个项目竣工投产，支持天马二期、宸美科技等80个项目增资扩产，新增20个智能制造试点示范项目。三是加快发展战略性新兴产业。牢牢把握未来产业竞争的主动权，大力发展新技术、新产品、新业态、新模式，培育壮大产业增长支柱。集成电路产业重点发挥联电、展讯、通富微电子等龙头企业作用，推进瀚天天成、华天恒芯等晶圆制造项目建设，促进一批重点IC设计项目落地。软件信息服务业重点扶持移动互联网、云计算、大数据、人工智能等细分产业发展，打造软件开发云和制造云。生物医药与健康产业重点加快建设企业协同创业中心，做强做精特宝生物、艾德生物等龙头骨干。新材料产业重点发展钨钼材料及合金、新能源电池、第三代半导体材料，建设石墨烯公共服务平台，培育10个应用示范工程，争创国家新材料产业集聚区。文化创意产业重点发展动漫游戏、创意设计、演艺娱乐等，办好中国-东盟电影节等影视节庆活动，策划生成一批时尚产业项目，确保产业链产值突破千亿。四是提质发展现代服务业。推动生产性服务业向专业化和价值链高端延伸、生活性服务业向精细化和高品质转变。加快建设丙洲、美峰现代服务业基地，提升发展9个省级现代服务业集聚示范区。建设前场、翔安等5个物流产业集聚区，深化国家现代物流创新发展和物流标准化等试点，打造进口商品集散中心和生产生活资料批发物流中心。加快创建国家全域旅游示范区，强化旅游市场监管，优化“吃住行”服务质量，提升“游购娱”经济效益。办好第20届投洽会等重要展会，规划建设第二会展中心，提升旅游会展业国际化、高端化、市场化水平。

　　（二）坚定不移推进跨岛发展，全力打造高颜值生态花园之城

　　着力构建城市发展大格局，继续推进岛内外一体化，统筹城市规划、建设、管理，不断增强发展的系统性持续性宜居性。一是加大岛外开发建设力度。坚持高起点、高标准、高层次、高水平，推动新城基地建设提速提质提效，促进城乡建设统一规划、产业合理布局、基础设施互联互通。建成投用滨海东大道等市政基础设施，加快双十中学翔安校区、翔安妇幼保健院等公共服务配套建设，确保集美新城161个项目完成投资200亿元、环东海域新城和现代服务业基地300个项目完成投资500亿元、马銮湾新城229个项目完成投资220亿元、同翔高新技术产业基地56个项目完成投资40亿元。二是实施乡村振兴战略。按照产业兴旺、生态宜居、乡风文明、治理有效、生活富裕的总要求，为乡村振兴提供全方位制度支持，吸引城市资本、技术、人才等要素更多向农业农村流动，推动农业全面升级、农村全面进步、农民全面发展。深化农业供给侧结构性改革，由增产导向转向提质导向，坚持质量兴农、绿色兴农，大力发展设施农业、特色农业、生态农业和远洋渔业，加快建设27个农业产业项目，促进一二三产融合发展。推动农村基础设施提档升级，建设30个美丽乡村，完成129个自然村污水集中纳管和346个自然村污水分散式处理。深化农村集体产权制度改革，推动资源变资产、资金变股金、农民变股东。落实农村土地所有权、承包权、经营权“三权分置”制度，稳定土地承包关系，大力推动经营权向农业龙头企业、农民专业合作社等流转。发展新型经营主体，促进小农户与现代农业发展有机衔接，让农民合理分享全产业链增值收益。三是加快完善基础设施。力争完成投资670亿元。围绕建设国际性综合交通枢纽，海港方面要加快国际航运中心软硬件设施配套；空港方面要力促翔安机场主体工程正式开工，加快临空产业经济区规划建设；轨道交通方面要完善地铁1号线运营服务，建成2号线车站主体结构并实现洞通，加快推进3、4、6号线建设，统筹地铁沿线综合开发；城市道路方面要完善“两环八射”快速路网，建成海沧货运通道，加快第二西通道建设，开建第二东通道，做好第三东通道等前期工作，规划建设进出岛通道公交换乘枢纽。围绕确保城市用水安全，建成西水东调新通道、汀溪水库群至翔安水厂等水源连通工程，推进九龙江北溪雨洪利用、进岛输水第二通道等前期工作。围绕确保城市用电安全，投运一批输变电项目，加快建设世界一流城市配电网。四是着力提升城乡品位。编制完善城市总体规划，启动岛内老城区保护性开发和旧城旧村改造更新，选择若干片区开展试点、逐步推开。大力推进城市精细化管理，改造提升园林绿化、建筑立面、市政设施、夜景照明，集中整治占道经营、油烟扰民、破墙开店、共享单车无序停放等问题。采取最坚决的整治措施，对存量“两违”限期拆除，对新增“两违”一律拆除。实施“厕所革命”行动计划，优化公厕布局，新建公厕90座以上。五是坚决打好污染防治攻坚战。深入推进生活垃圾分类，岛内全面实现精准规范，海沧、集美覆盖率实现80%，同安、翔安覆盖率实现70%，焚烧处理率提高到50%以上，加快建设厨余垃圾处理厂和垃圾焚烧发电厂二期。积极创建国家生态园林城市，加强城市扬尘、海港空港、机动车辆、挥发性有机物等污染治理，确保空气质量保持全国前列；严格落实河长制，加强小流域截污控源，加快实施筼筜湖、五缘湾雨污分流，提标改造8个污水处理厂，开工建设马銮湾、西柯污水处理厂。坚持陆海统筹，严控入海污染物，彻底清退海域养殖。强化土壤污染管控和修复，依法严打小电镀，严防重金属污染。出台或落实53项生态文明体制改革成果，加快建设国家生态文明试验区。

　　（三）坚定不移实施项目带动战略，全力扩大有效投资

　　落实促进有效投资正向激励机制，充分发挥投资对优化供给结构的关键性作用，进一步提高投资有效性。一是扩大投资规模。加大招商引资力度，加强招商政策集成，以跑破脚皮的干劲、磨破嘴皮的韧劲，一抓到底，千方百计跑项目引资金。围绕产业链关键产品和项目，催生一批补链、强链、扩链配套项目；围绕产业链部署创新链、完善资金链，推进三链协同，切实提高实体经济竞争力。进一步降低民间投资门槛，放宽准入领域，实施一批政府与社会资本合作项目，不断扩大民间投资规模。二是推动项目滚动接续。完善“五个一批”项目推进机制，围绕国家产业政策、重大规划、重点投向，加强项目策划，持续生成一批重大重点项目。确保电气硝子、华润中心等102个亿元以上项目建成投产；大力扶持九牧厨卫、京东等53家高成长型企业发展；力争厦钨稀土永磁电机产业群等项目尽快落地。三是强化投资服务保障。落实重点项目一周一协调、一月一调度、一季一督查，完善联合会商、现场调度机制，突出签约、开工、投产三个关键环节，做到良性接续不断档。科学统筹用地、用林、用海、环境容量等指标，加快推进征地拆迁，集中清理批而未供、供而未用土地。更好运用财政资金、政府债券、城市发展基金支持项目建设，严格投资审核和工程决算。加大“晒”的力度，晒任务、晒项目、晒进度、晒成效，切实提高项目的开工率、建成率，把嘴上纸上项目变成地上项目。

　　（四）坚定不移深化改革开放，全力增强发展动力活力

　　继续推出一批标志性改革措施，着力推动更高水平的内外联动和双向开放，努力在全国发展大局中走前列、作示范。一是深化供给侧结构性改革。坚持去产能、去库存、去杠杆、降成本、补短板，促进生产要素从低质低效领域向优质高效领域集聚。坚决砍掉不合理行政事业性和经营性收费，维持较低社保费率水平，在降低企业税费、制度性交易、人工、融资、用能、物流成本等方面进一步减轻企业负担。深入推进“放管服”改革，复制推广自贸区工程建设和市场准入领域审批制度改革经验，推动社会民生、公共服务等领域审批服务事项取消、下放及调整。深入推进多证合一，强化事中事后监管，实现“双随机一公开”监管全覆盖。加快社会信用体系建设，完善市场主体诚信档案和市场退出机制。梳理公布更多“一趟不用跑、最多跑一趟”清单，完善线上线下服务大厅，增加24小时自助服务事项，推进全程网办、全城通办。二是大力推进重点领域改革。落实税收征管体制改革，合理划分财政事权和支出责任。加强跨部门和内部资金统筹使用，集中财力办大事，提高财政专项资金使用效率。坚持以管资本为主，推进国有资产集中统一监管、优化布局，提高配置效率。分类推进国企改革，发展混合所有制经济，鼓励员工持股，开展专业化整合重组。坚持有进有退、聚焦主业，支持国企投资新兴产业和高新技术产业，培育更多具有国际竞争力的一流企业。三是用好自贸试验区更大改革自主权。对照最高水平、最高标准，大胆试、大胆闯、自主改，不断推进制度创新和复制推广。拓展国际贸易“单一窗口”功能，进一步优化监管，提升跨境贸易效率。充分发挥“保税+”、“互联网+”、“金融+”等功能优势，继续推进一批重点平台建设，加快一站式航空维修基地建设，大力发展融资租赁等新兴业态。进一步加强对建设中国特色自由港战略构想的探索。四是打造“海丝”支点城市。争取厦门过境口岸资质，力争实行7×24小时货物通关运营，放大中欧（厦门）班列辐射带动作用，构建“陆丝”和“海丝”无缝对接的重要陆海空枢纽城市。加大对“金砖+”和“海丝”沿线国家的招商力度。巩固东盟传统贸易市场，加快建设重点商品出口基地，扩大双向贸易规模。依托中国-东盟海洋合作中心等平台，推动海洋产业、科技、文化等领域合作。发挥侨务优势，用好侨资侨智。五是提高对外合作水平。落实国家外商投资产业指导目录，放宽相关领域外资准入限制。积极争取国家跨境电商综合试验区试点，加快发展外贸综合服务等新型贸易业态。支持企业拓展境外业务，带动相关技术、成套装备和服务贸易出口，加大紧缺资源性大宗商品进口。积极推动厦漳泉龙同城化，深化闽西南五市、闽粤赣十三市区域合作，加强与甘肃、宁夏等东西部扶贫协作，落实援藏援疆援渝等任务。

　　（五）坚定不移深化融合发展，全力打造对台交流合作战略支点

　　认真贯彻中央对台大政方针，更好发挥对台工作综合优势，为促进两岸关系和平发展作出更大贡献。一是提升经贸合作实效。加快建设“一区三中心”，深化先进制造业和现代服务业对接合作，鼓励台资银行、证券、保险等金融机构来厦设立总部和资金营运中心。争取率先在自贸区落实海峡两岸服贸协定若干对台开放措施，建设两岸检验检疫合作试验区。加快建设五通客运码头三期和海峡旅游服务中心，争取赴金旅游便利化政策，培育厦金精品旅游线路，打造厦金融合发展示范区。二是密切亲情纽带联系。精心筹办第十届海峡论坛，持续提升文博会、图交会、民间艺术节等活动实效和品牌效应。打造一批国家级海峡两岸青创基地、对台研学旅行基地，吸引更多台湾青年来厦就业创业。积极研究推动在厦台胞享受市民待遇、台企享受陆企待遇，增进同胞福祉，促进心灵契合。

　　（六）坚定不移补齐民生短板，全力提升市民获得感幸福感

　　紧紧抓住人民最关心最直接最现实的利益问题，着力办好10大类为民办实事项目，推进城乡基本公共服务均等化，让改革发展成果更多更公平惠及全市人民。一是优先发展教育事业。落实立德树人根本任务，发展素质教育，推进教育公平。加强师德师风建设，提高教师待遇，探索推行校长职级制，加大名校长名师培养力度。推动学校与新城、幼儿园与住宅同步规划、同步建设、同步使用，建成20个中小学项目，开工20个幼儿园项目，新增学位2.75万个。支持国企举办普惠性幼儿园，试点开展公办幼儿园、小学延时服务。推进名校跨岛发展，建成投用厦门一中海沧校区、科技中学翔安校区，提升城乡义务教育一体化水平。强化随迁子女积分入学政策导向。鼓励中外合作办学，建设优质民办国际学校。加快高等教育发展，支持一流大学和一流学科建设。加快二元制、现代学徒制、应用型职业教育发展。二是加快建设健康厦门。开工建设马銮湾医院，建成投用翔安医院、市心血管病医院、弘爱医院等重大项目和一批社区卫生服务中心，新增床位4000张。高位嫁接高端医疗资源，更好发挥复旦大学中山厦门医院、北京中医药大学厦门医院引领带动作用，推动集美新城医院合作项目落地。鼓励社会资本举办高端护理、妇儿等特色专科。深化公立医院改革，正式运营医管中心，完善医疗急救体系运营管理，加强基层全科医生队伍建设。深入开展爱国卫生运动，倡导健康文明生活方式。加强计生服务管理，做好政策配套衔接。三是繁荣发展文体事业。弘扬中华优秀传统文化，培育和践行社会主义核心价值观。持续做好鼓浪屿世界文化遗产保护利用。完善公共文化服务体系，扶持艺术精品创作，推进图书馆、文化馆、闽南戏曲艺术中心建设，加快建设闽南文化生态保护实验区。广泛开展全民健身活动，统筹规划体育场馆，有序推动学校、机关、企事业单位体育设施对外开放。做优国际品牌赛事，做大体育产业规模，打造区域性赛事中心城市。四是完善就业社保体系。针对被征地农民、海域退养渔民、高校毕业生等重点群体，做好就业创业服务和技能培训。办好线上线下招聘会，满足企业用工和人才需求。健全城乡居民医保筹资和重特大疾病保障机制，提高低保对象和特困人员救助供养标准。加强社保基金安全管理，完善监管体系。建设39家农村幸福院和社区日间照料中心、15个智慧养老示范社区，引导社会资本投向医养护结合的高端养老产业。

　　（七）坚定不移推进安全发展，全力维护社会和谐稳定

　　把防风险摆在突出位置，加强各类风险预警研判和隐患排查治理，提高防范化解和应对处置能力，牢牢守住安全发展的底线。一是加强金融、房地产等风险防控。加大对金融风险的防范力度，加强对融资担保、小额贷款、区域性股权中心、商业保理等地方金融机构的监管，开展互联网金融风险专项整治，严厉打击非法集资等违法违规行为，控新化旧压降不良贷款，确保不发生系统性金融风险。全面建设国家产融合作试点城市，完善政策性担保体系，推动政银企对接常态化，大力支持236家后备企业上市，提升服务实体经济水平。加强政府性债务风险防控，用好用足置换债券额度，优化期限结构，腾出更多资金用于重点项目建设。加强企业债务风险防控，及时化解隐患，用足用好核销政策，特别要严控国有企业存贷风险、应收账款风险和现金流风险，确保资金链运转安全顺畅。健全完善房地产市场调控长效机制，推进租购并举住房制度建设，实行差别化的调控政策，满足首套刚需，支持改善需求，有效引导市场预期，加强商品住宅土地供应，加快商业地产去库存，确保去化周期和房价控制在合理区间，全面加快地铁社区等保障性住房建设，促进房地产市场平稳健康发展。二是提升公共安全水平。严格落实安全生产责任，全面排查治理安全隐患，坚决防范重特大事故发生。完善“从农田到餐桌”全链条监管，让市民吃得放心，确保建成国家食品安全示范城市。加强粮食工作，保障粮食安全。完善基层防汛抗台、防灾减灾体系，加强危房、地灾隐患点整改。持续推进平安厦门建设，健全城市公共安全治理体系，提升治安防控社会化、法治化、智能化、专业化水平。三是加强和创新社会治理。完善城乡社区治理，提高网格化管理效率。做好第四次全国经济普查。确保村居换届选举顺利完成。支持工青妇等群团组织深化改革。加大社会组织培育监管力度，完善政府购买社工服务机制。推动文明城市创建继续在全国走前头、立标杆。深化军民融合，巩固提升双拥共建水平。运用法治思维和法治方式处理信访问题，妥善解决群众合理合法诉求。

　　三、全力建设人民满意的服务型政府

　　深入学习贯彻习近平新时代中国特色社会主义思想，牢固树立“四个意识”，坚定“四个自信”，坚决维护以习近平同志为核心的党中央权威和集中统一领导，在政治立场、政治方向、政治原则、政治道路上同以习近平同志为核心的党中央保持高度一致，不折不扣地把党中央国务院、省委省政府和市委各项决策部署落到实处。

　　加强政府系统党的建设。落实全面从严治党要求，严格遵守政治纪律和政治规矩，严格执行新形势下党内政治生活若干准则。认真开展“不忘初心、牢记使命”主题教育。坚决按照《厦门市进一步贯彻落实中央八项规定精神执行办法》，驰而不息整治“四风”。严查重点领域和关键岗位腐败，严惩群众身边腐败问题，促使广大公务人员心存敬畏、手握戒尺、干净干事。

　　强化责任担当。全面增强“八种本领”，完善政府决策、责任落实、督查问责机制，以钉钉子精神把各项工作落细落小落实。弘扬“马上就办、真抓实干”作风，大力提振干部精气神，既雷厉风行又久久为功，真正做到想干事、能干事、干成事。坚持严管和厚爱、激励和约束并重，旗帜鲜明为那些敢于担当、踏实做事、不谋私利的干部撑腰鼓劲，让干得好的牛起来、干不好的坐不住。

　　推进依法行政。自觉接受市人大及其常委会法律监督、工作监督和市政协民主监督，健全与人大代表、政协委员的沟通联络机制，高质量办好人大代表议案、建议和政协委员提案。坚持厉行法治，深化全民普法，提高政府立法质量，强化重大行政决策合法性审查，严格规范公正文明执法，提升法治政府建设水平。推进政府决策、执行、管理、服务、结果“五公开”，加强对权力运行的制约和监督，增强政府公信力和执行力。

　　优化政务服务。坚持问题导向，对标国际一流，全力营造法治化、国际化、便利化的营商环境。加快政府职能转变，坚决扫除经济发展的体制机制障碍。全面整治庸政懒政怠政，构建亲清新型政商关系，坚决纠正推诿扯皮、办事拖沓等行为，使政府服务更贴心，让市民办事更省心。

　　各位代表！幸福靠奋斗，爱拼才会赢。让我们更加紧密地团结在以习近平同志为核心的党中央周围，在省委省政府和市委的坚强领导下，不忘初心、牢记使命，凝心聚力、锐意进取，加快建设“五大发展”示范市，勇当新时代中国特色社会主义排头兵，奋力谱写新时代厦门发展新篇章!
